# A call to integrate menstrual cycle influences into just-in-time adaptive interventions for suicide prevention

**DOI:** 10.3389/fpsyt.2024.1434499

**Published:** 2024-12-05

**Authors:** Hafsah A. Tauseef, Daniel D. L. Coppersmith, Azure J. Reid-Russell, Anisha Nagpal, Jaclyn Ross, Matthew K. Nock, Tory Eisenlohr-Moul

**Affiliations:** ^1^ Department of Psychiatry, University of Illinois Chicago, Chicago, IL, United States; ^2^ Department of Psychology, Harvard University, Cambridge, MA, United States

**Keywords:** just-in-time adaptative intervention, menstrual cycle, suicide, idiographic modeling, mobile health, digital phenotyping, passive measures, self-injury

## Abstract

This paper discusses the scientific rationale and methodological considerations for incorporating the menstrual cycle as a time-varying intra-individual factor in personalized medicine models, such as Just-In-Time Adaptive Interventions (JITAIs). Among patients, accumulating evidence suggests that the normal hormone fluctuations of the menstrual cycle represent a time-varying factor that can trigger or exacerbate psychiatric symptoms, including but not limited to affective dysregulation, suicidality, and irritability. While only a minority of the general female population experiences significant cyclical changes, this hormone-sensitive response appears to be greater among patients with psychiatric disorders, with studies demonstrating that a majority of patients recruited for past-month suicidal ideation demonstrate worsening of their suicidality around menses. However, no interventions target suicidality during this monthly period of elevated risk despite evidence of a clear recurring biological trigger. This unique and recurrent “biotype” of suicidality is well-suited for JITAIs. In addition to providing a rationale for the inclusion of the cycle in JITAI, we provide illustrative options and examples regarding the measurement and implementation of cycle variables in JITAIs. We discuss how JITAIs might be leveraged to use menstrual cycle data to identify states of vulnerability within people and strategically select and deploy interventions based upon their receptivity at various phases in the cycle. Furthermore, we discuss how to integrate passive measures for tracking the menstrual cycle. Although much research is needed before implementation, we maintain that the menstrual cycle represents a critically understudied time-varying feature that may markedly improve the accuracy of JITAI models for predicting suicidality.

## 1 Introduction

Despite decades of research, suicide deaths in the United States have not decreased, with mortality rate increasing by 35% between 2000 and 2018 (1). Nonetheless, empirical studies have yielded a critical insight: suicidal ideation (SI) and behaviors (SB) result from highly complex processes that fluctuate over time in person-specific (i.e., idiographic) ways (2, 3). In response, scientists are developing Just-in-Time Adaptive Inventions (JITAIs) for suicide prevention, which model specific time-varying risk factors for each person and intervene in timely, tailored ways (4). In tandem, longitudinal and experimental studies have begun to establish the menstrual cycle as a critical time-varying source of imminent suicide risk, especially in those with chronic suicidality (5–8). Responding to calls from the National Institute of Health to (a) use JITAIs to optimize mental health treatments (9) and (b) focus on female-specific health conditions (10, 11), we argue for integrating the menstrual cycle into JITAI development for suicide prevention. This manuscript discusses why JITAI models may be optimal for treating menstrual cycle-related suicidality, methodological recommendations, and considerations for future research.

### 1.1 Brief introduction to digital interventions and JITAI

Digital interventions broadly involve identifying vulnerability (e.g., adverse health symptoms) and receptivity [e.g., readiness to use supports (12)] states to deploy personalized interventions aimed at reducing both proximal (i.e., mediator pathways) and distal (i.e., ultimate clinical goals) outcomes. JITAIs tailor treatment type, timing, and intensity based on a patient’s evolving needs, delivering support when it is most effective, and patients are most receptive (13). Typically delivered via smartphone, JITAIs have been studied across various health conditions (14–16). They involve decision points (when interventions can be delivered), intervention options (treatments available at decision points), tailoring variables (when and how to intervene), and decision rules (guidelines for choosing and timing interventions).

### 1.2 JITAI is a promising method for personalized suicide prevention

There is growing interest in applying JITAI to psychopathology research and practice, including suicidality (4, 17, 18), given the dynamic nature of mental health symptoms (19, 20). JITAIs may enhance evidence-based treatments for mental health conditions (4), for example, by promoting coping skill use during critical moments in between psychotherapy sessions. Specifically, JITAI aims to deliver the best-suited evidence-based intervention for an individual at any given moment based upon the individual’s previous data. While there are no published studies on JITAIs for suicidality, researchers are currently theorizing the best methodology (regarding feasibility, statistics, and ethics) for building these models (2, 4). This work builds on previous suicide research, indicating that suicidality is (a) heterogeneous, with no singular antecedent, content, or function (2, 21), and (b) time-varying (3), and that (c) mobile and internet interventions for suicide prevention show positive treatment effects (22). Thus, JITAI is a promising method in development for suicide prevention.

### 1.3 The menstrual cycle as a powerful idiographic predictor of suicide risk and associated symptoms for JITAI models

While suicide death is more common among males, females exhibit a greater risk of depression, SI, and suicide attempts—particularly during reproductive years, when ovarian hormones are elevated and fluctuating (23, 24). The menstrual cycle is the primary source of these fluctuations, responsible for a predictable monthly pattern of ovarian hormone change. While most females do not experience significant affective changes in response to the cycle, a substantial minority experience distressing symptoms requiring diagnosis and treatment (25, 26). The menstrual cycle represents a recurring time-varying risk factor for imminent suicide risk in some—but not all—females. Cross-sectional studies demonstrate that patients are more likely to make a suicide attempt just prior to and during menses (27, 28). Among females recruited for past-month SI, most patients demonstrate peak affective symptoms and SI around menses onset, with the cycle accounting for approximately 25% of the within-person variance in daily SI (7, 8). Similarly, suicide risk is elevated among patients with prospectively-confirmed premenstrual dysphoric disorder (PMDD)—a severe form of emotional premenstrual symptoms (29). In a global study of PMDD patients, 71.6% indicated lifetime active SI, 48.58% planning, and 34.72% reported lifetime attempt. A smaller, more tightly controlled study observed high rates (~40%) of current SI in the luteal phase of patients with prospectively-confirmed PMDD (29).

The menstrual cycle is not only a crucial biological predictor of suicide risk but also of suicide-related symptom networks that can guide targeted interventions. Recent work has identified several affective symptoms (e.g., depression, perceived burdensomeness) as mediators of the relationship between the menstrual cycle and suicidality (8). Additionally, distinct hormonal events within the menstrual cycle trigger different symptom clusters associated with suicidality. For instance, progesterone surges in the luteal phase are linked to irritability, interpersonal conflict, and hyperarousal, while estrogen withdrawal before and during menses is associated with depression, anhedonia, and impaired cognitive function (6, 28). These symptom profiles—one driven by progesterone and the other by estrogen—provide a framework for understanding how menstrual cycle phases can correlate with specific patterns of suicidality and pave the way for JITAI models to match hormone-driven suicide-symptom clusters to specific interventions that deploy at the appropriate time in the cycle.

**Figure 1:**
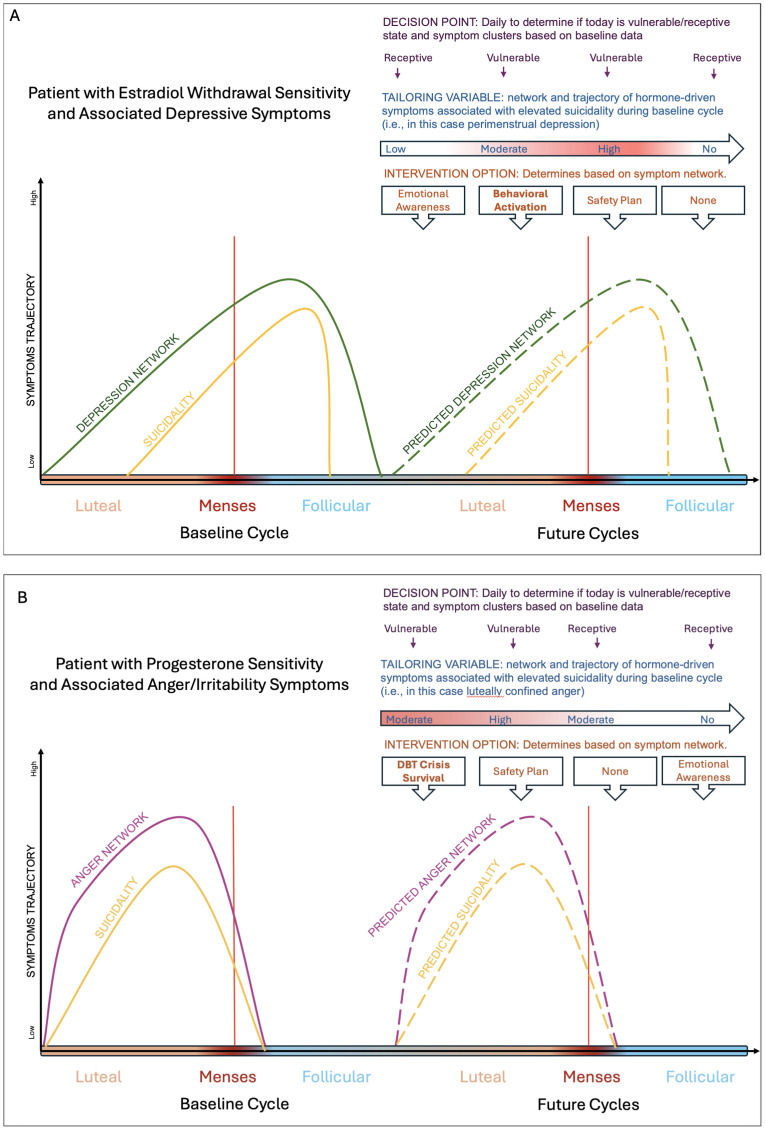
Schematic figure depicting how JITAIs can be used to identify risk states and interventions for patients who experience changes in their suicidal thoughts and behaviors across the cycle from their baseline ratings. Panel **(A)** is a schematic of a patient experiencing premenstrual exacerbation (i.e., worsening of their symptoms around menses onset) of their psychiatric symptom, with the peak worsening a few days after menses onset. The idiographic driver of their suicidal thoughts and behaviors worsening is depression and hopelessness. In this case, behavioral activation could be deployed to mitigate depression and hopelessness. Panel **(B)** is a schematic of a patient experiencing luteal confined symptoms, with the symptoms worsening a few days prior to menses onset and completely clearing out a few days post menses. The idiographic driver of their suicidal thoughts and behaviors is anger and interpersonal conflict. In this case, skills from Dialectical Behavioral Therapy may be deployed such as crisis survival skills may be deployed.

In summary, the luteal/perimenstrual phase of the menstrual cycle may represent the only recurrent, biological, predictable, idiographic variable capable of predicting phases of increased suicide risk and associated symptom clusters. Incorporating the menstrual cycle into JITAI models offers the opportunity to better predict when an individual is most likely to experience suicidality and which hormone-driven affective symptom cluster is most linked to suicidality for the individual, all in service of delivering timely and effective evidence-based treatments (6, 29, 30). In **Figure 1** we review a schematic model of how the menstrual cycle may be leverged for JITAI models.

### 1.4 The implementation of JITAI is useful for scientific and treatment research on menstrual cycle-related psychiatric changes

Currently, the only cycle-related psychiatric diagnosis is PMDD, which affects 5.5% of females (31) and is characterized by significant affective, cognitive, and physical symptoms during the week prior to menses that become minimal or absent by the time menses ends. However, similar cyclical changes appear to be more prevalent among psychiatric patient populations, termed *premenstrual exacerbation (PME);* in which psychiatric symptoms persist throughout the cycle but worsen premenstrually (32). PME has been observed frequently among those with depression (31), suicidality (8), and other psychiatric disorders, including borderline personality disorder, eating disorders, and bipolar disorder (33, 34). However, there is currently no DSM-V diagnosis or specifier for PME. Although these clinical categories (i.e., PMDD/PME) are helpful in specific clinical contexts, there is more variability and dimensionality in symptoms across the cycle than can be captured by these trait-like diagnoses.

Integrating JITAI with the menstrual cycle addresses methodological challenges of high false positive rates in cross-sectional assessments of premenstrual symptom change (34, 35)]. Moreover, JITAI’s reliance on ecological momentary assessment (EMA) allows for a baseline assessment in which symptoms are rated daily across two menstrual cycles to inform the timing of future assessment and intervention. JITAI also allows for a dimensional/continuous approach to modeling affective and suicidal symptoms across the cycle—without being hindered by the need for discrete diagnostic categorization (i.e., PMDD/PME).

Most importantly, JITAI approaches align with the unmet treatment needs of patients with cyclical affective symptoms (i.e., PMDD/PME) and suicidality. JITAI’s person-specific data collection and modeling are well-suited to support understanding the role of the menstrual cycle in patients’ unique symptom trajectories, which is critical given the significant heterogeneity in the timing and content of premenstrual symptoms (30, 36). Therefore, JITAIs seem well-suited to enhance treatment for this population, given the limited number of effective treatment options, limited expert providers, and the strong patient desire for psychosocial interventions (37).

## 2 Preliminary methodological recommendations for integrating menstrual cycle-related suicidality in JITAI development

Idiographic suicide research has been primarily qualitative (i.e., suicide notes, case studies, and chart reviews) rather than quantitative due to statistical constraints. However, computational advancements (e.g., machine learning) enable more complex modeling that accommodates the heterogeneity of suicide processes across time and unique to each patient (21, 38, 39). We argue that JITAIs can predict how cycle phase drives certain symptoms that exacerbate suicidality and then select the appropriate intervention based on those hormone-driven symptoms. Below we describe how menstrual cycle data can be used in a variety of ways as a digital biomarker (40).

### 2.1 Selecting patients

JITAI for menstrual cycle-related suicidality is designed for patients experiencing predictable symptom changes across their natural menstrual cycle and patients with PMDD. Thus, the first prerequisite is selecting individuals who are “naturally cycling” without additional hormone variability [e.g., not pregnant, on hormonal birth control, or perimenopausal; for further details see (41)]. Next, this naturally cycling patient must demonstrate menstrual cycle-related suicidality; thus, at least two months of baseline symptom ratings are needed. This will allow for accurate modeling of the typical timing of symptom onset relative to the percent of cycle phase elapsed and symptom clusters. Alternatively, if patients already have prospectively confirmed PMDD or PME of SI diagnoses from a provider, they may benefit from this JITAI as well.

### 2.2 Statistical models for identifying subgroups of individuals for whom the menstrual cycle significantly impacts suicidality

Currently, algorithms for assessing PMDD and PME are available [C-PASS (42] although they remain strict in their diagnostic categorization, similar methods could be applied flexibly to understand cyclical change. As an initial approach, one might compare the mean of the highest-risk premenstrual days (3 days prior to menses onset to the first two days of menses) to the mean of the lowest-risk postmenstrual days (days 6-12 after menses onset) and evaluate if there is >=15% change in suicidality (8, 42). However, other statistical methods could be applied, as well, see (**Table 1**).

**Table 1:** Identification of individuals with Menstrual Related Suicidality Changes and their discrete risk states across the menstrual cycle.

Pre-Assessment: Determining subgroups of people and finding their states of vulnerability.	
*Examples of statistical models used to examine subgroups of people based on menstrual cycle-dependent symptom variability; using menstrual cycle-dependent symptom data as a diagnostic biomarker* (40)	*Examples of statistical models used to determine vulnerable states across the menstrual cycle; using menstrual cycle data as a monitoring biomarker* (40) *to identify high-risk states*
**Latent Class/Profile analysis** (50): models for understanding if distinct subgroups of participants, based on both concurrent symptoms and menstrual cycle variables, predict engagement in suicidality.	**Multilevel Models** (41, 51): can use spline models and place knots at biologically relevant timepoints such as menses onset or ovulation to determine rate of change between knot points. Polynomials can also be used to flexibly model nonlinear change across the cycle.
**Group-based trajectory modeling** (36, 52, 53): non-linear applications can be used to subgroup patients based on how suicidality changes over the menstrual cycle, which may support understanding which participants experience significant variability	**Hidden Markov models** (54, 55): machine-learning approach for finding discrete states of suicidal risk within a series of menstrual-cycle and concurrent symptoms across time, as well as finding the probability at a given time-point of transitioning between high-risk and low-risk states.
**Group Iterative Multiple Estimation Model** (21, 39, 56): creates networks of symptoms and shared paths for each patient and then subgroups patients based on shared networks. The menstrual cycle can be added as an exogenous variable to determine if it meaningful contributes to patient’s suicide network.	**Individual Regression models:** apply regression models to an individual’s menstrual cycle and concurrent symptoms time series data to predict engagement in suicidality.
**Carolina Premenstrual Assessment Scoring System (C-PASS)** (42): algorithms developed based on DSM-5 PMDD diagnosis and PME; can compare the maximum mean of the high-risk premenstrual days (7 days to 1 day prior to menses onset) and the minimum mean of the low-risk postmenstrual days (4 days to 10 days after menses onset) and see if there is at least a 15% change in suicidality symptoms	**Vector Autoregression Models** (57, 58): models applied to an individual’s data that may be used to understand how menstrual cycle variables may dynamically associate with concurrent symptoms or suicidality over time (cross-lagged effects) and how menstrual cycle, psychopathology, or suicidality variables may persist over time (autoregressive effects).

### 2.3 Measuring the menstrual cycle, using wearable technology to detect cycle status, and identifying risk states

There are various ways to measure the menstrual cycle in service of identifying vulnerable states. Given the variability in cycle lengths between and within individuals (43, 44), baseline cycles’ menses onset and ovulation dates can be used to standardize time across the menstrual cycle, providing the percentage of cycle (or cycle phase) elapsed rather than days of the cycle. This approach enhances the precision of continuous modeling strategies in identifying future risk windows, aligning more accurately with ovarian hormones and symptom changes.

When only menses onset dates are available, the duration of each cycle is calculated from one menses onset to the day before the subsequent menses. This duration can then be used to determine the percentage of the cycle elapsed. This percentage is mapped onto subsequent cycles to predict days of increased risk. For example, if a patient has cycles of 32 and 30 days, and symptoms worsening or onsetting at day 28 and 26, respectively, corresponding to 87.5% and 86.7% of their cycle, future cycles may have an estimated duration of 31 days, with symptom onset at approximately 87% of the way through the cycle.

When both menses onset and ovulation dates are available, the luteal phase duration can be calculated from the day after ovulation to the day before the next menses onset, and the follicular phase from menses onset to estimated ovulation (from which percentage of each phase elapsed can be determined for each day). For instance, if a patient’s first cycle has a 14-day follicular phase and a 12-day luteal phase, with symptom onset 10 days into the luteal phase (83.3% elapsed), and the second cycle has a 15-day follicular phase and an 11-day luteal phase with symptoms onset 9 days into the luteal phase (81.8% elapsed), these percentages can inform future risk predictions.

Wearable and smartphone technologies can also be used to track menses dates and identify ovulation. Cycle-tracking applications, such as Apple^®^ Health, collect daily self-reported data on menses status (bleeding vs. not bleeding) and use proprietary algorithms to forecast future menses start dates (45), with a study indicating 40% of the assessed cycles had daily recordings (46). Wearables monitor physiological functions impacted by the cycle, such as thermoregulation and heart rate variability. A high-specificity method for passively detecting ovulation is a wearable tracking basal body temperature (BBT). Progesterone’s thermogenic effect causes an abrupt (surpassing 37°) and sustained temperature elevation in BBT, indicating ovulation. Another potential indicator is heart rate variability (HRV), which is downregulated in the luteal phase, correlating inversely with progesterone (47, 48). Studies are underway to validate passive HRV measurement to determine cycle events (49). Wearables (e.g., Apple Watch^®^, FitBit^®^, Oura Rings^®^) that can passively detect either BBT, HRV, or both would significantly reduce participant burden by passively detecting ovulation. However, for validation of these approaches, it may be prudent for cycle-focused studies to initially include urine luteinizing hormone (LH) testing to evaluate the occurrence and timing of ovulation (LH surges and peaks prior to ovulation), as described in (41).

### 2.4 Identifying discrete vulnerability states across the menstrual cycle for suicidality based on person-level fluctuations

Once patients are identified as having menstrual cycle-related suicidality (or PMDD/PME), we may pinpoint their vulnerability states throughout the cycle. Group studies suggest peak suicidality occurs three days before and two days after menses onset (7). For JITAI, various statistical models (see **Table 1**) can identify specific times of suicidality risk based on menstrual cycle time elapsed. These vulnerable states should be stratified into “no”, “low”, “moderate”, and “high” risk days. Interventions can be tailored based on symptom networks exacerbating suicidality.

### 2.5 Proximal and distal outcomes

We propose person-specific, symptom clusters that mediate the cycle-suicide relationship as the proximal outcome in our example JITAI. The aim of the example JITAI is to deploy interventions that reduce these monthly states of increased hormone-driven symptoms, in order to prevent future suicidality, our distal outcome.

### 2.6 Decision point

The daily decision point for this JITAI will identify if today is a vulnerable day in the cycle (i.e., a day associated with elevated suicide risk based on baseline cycles). While we anticipate predictable trajectories for SI and SB, we currently recommend daily self-report assessment of suicidal symptoms. However, future JITAI research may move beyond self-report with the development of valid, passively collected prognostic biomarkers of suicide risk, such as smartphone typing patterns (59), fewer phone calls and messages (indicating social withdrawal), and so on.

### 2.7 Tailoring variables

The tailoring variable for this JITAI is the cluster of symptoms driving perimenstrual worsening of suicidality for the individual. Individual baseline data is used to define cycle days associated with increased suicide risk as well as symptom networks that drive this increase in suicidality (e.g., anger/conflict in response to a progesterone surge, depression/anhedonia in response to estrogen withdrawal).

### 2.8 Intervention options and personalization

We propose using network models from baseline data to tailor interventions based on individual symptom clusters. JITAI holds the potential to match evidence-based interventions to distinct symptom clusters linked with hormone-driven suicidality within an individual. To illustrate: on risky cycle days, individuals may benefit from receiving a reminder to increase awareness of this vulnerable cycle phase (e.g., “You are at a point in your cycle when depression, hopelessness, and guilt worsen, increasing SI risk”). As risk increases, luteal-phase irritability and affective lability could be targeted with skills from Dialectical Behavior Therapy (e.g., TIP, opposite action to anger). Meanwhile, perimenstrual depression could be targeted with behavioral activation.

Micro-randomized control trials, a type of clinical trial where each relevant decision point is randomly assigned within each person (60), are needed to identify optimal treatments and receptivity states for menstrual cycle-related suicidality and co-occurring affective symptoms. Although current research does not specify receptivity states across the menstrual cycle, we hypothesize that patients may be more receptive to learning and applying skills during non-risky days (e.g., follicular phase). Adapting frameworks such as the multiphase optimization strategy (MOST), which involves a screening, refining, and confirming phase of building a digital interventions, which allows examination of individual components of intervention and delivery (61) may be helpful.

Patients may opt to take their baseline rating to their providers for psychoeducation and diagnosis of PMDD, if applicable. For patients with PMDD, selective serotonin reuptake inhibitors (SSRIs) are an effective treatment option, with some patients seeing symptom reduction within 24-48 hours of taking their medications (62). SSRIs for PMDD patients are prescribed either continuously or to be taken just in the luteal phase (62). For patients who are taking a luteal phase-only SSRI regimen, JITAI can send daily scheduled reminders for medication adherence starting the first day post-ovulation.

### 2.9 Decision rules

In the schematic decision rule below, we present an example of an operational system that increases participant awareness of their risky phase and deploys interventions well-matched to suicide-related symptoms during their specific vulnerable state.

If the baseline maximum risk score for this menstrual cycle day number is “no risk”:

                      Then: “No message”

           Else if baseline risk score for this menstrual cycle day number is “low risk”:

                      Then: “Send auto-message that they are entering a vulnerable window in their cycle and recommend non-judgmental awareness of emotions”

           Else if baseline risk score for this menstrual cycle day number is “moderate risk” OR “high risk:

                      Then: “Send patient tailored skill based on the symptom network (e.g., behavioral activation for depression related to estrogen withdrawal or DBT skills for anger/mood swings related to progesterone surge”

## 3 Discussion

For many patients, the menstrual cycle is a recurring biological predictor of suicide risk. However, there is currently no evidence-based treatment for menstrual cycle-related suicidality. Given the complex, idiographic, and dynamic impact of the menstrual cycle on suicidality, we propose that JITAI are a promising avenue for further exploration. Integrating menstrual cycle variables into JITAI models can leverage a biological variable to predict when and which symptoms are exacerbating suicidality, with the goal of deploying timely, effective interventions. While self-report is a key component of currently proposed models, the advancement of wearable and smartphone-based cycle tracking technologies may allow for low-burden, passive measurement in the future.

### 3.1 Challenges and future directions

While JITAIs offer promise in addressing menstrual cycle-related suicidality, there are distinct challenges in their development and implementation. First, atypical menstrual cycles, related to anovulation, contraceptive use, peripuberty, or perimenopause (63), pose obstacles to accurate prediction of risky timeframes. Thus, research should account for nuances in cycle variation. Ethical concerns arise regarding intervention timing and methods for high-risk patients. Clear protocols must address imminent risk, balancing intervention efficacy with confidentiality and potential need for hospitalization. Although in this hypothesized model, daily variables were used for tailoring variables, we hope that eventually, suicide and menstrual research will move past daily measurement to more within the moment (i.e, hourly to seconds) detection of states of vulnerability and receptivity, allowing for more timely responses to perimenstrual suicide changes. Finally, before the deployment of JITAI for menstrual cycle-related suicidality, randomized controlled trials are needed to determine (a) which cycle-timed interventions are most acceptable to patients, (b) when patients are most receptive to interventions (12), and (c) whether targeting monthly risk windows with JITAI reduces future suicide attempts. Finally, while our manuscript primarily addresses menstrual cycle-related psychiatric changes, we acknowledge that the menstrual cycle has an impact on broader biological systems [i.e., migraines and epilepsy (64)] and emphasize that the broader digital therapeutic research accounts for menstrual-related changes.

## 4 Conclusion

We advocate for integrating the menstrual cycle into JITAI models for suicide prevention to address individual variability in suicidality and psychiatric symptoms. Despite implementation challenges, we propose including the menstrual cycle as a variable in JITAIs, particularly for suicide prevention.

## Data Availability

The original contributions presented in the study are included in the article/supplementary material. Further inquiries can be directed to the corresponding author.
